# 本科生综合实验：基于卤键有机框架材料的分散固相萃取方法构建及在溴代阻燃剂分析中的应用

**DOI:** 10.3724/SP.J.1123.2026.04016

**Published:** 2026-10-08

**Authors:** Dandan WANG, Jiaqi HU, Cui QIN, Mingyue WANG, Famin KE, Hong NIU, Xilin YANG, Die GAO

**Affiliations:** 西南医科大学药学院，四川 泸州 646000; School of Pharmacy，Southwest Medical University，Luzhou 646000，China

**Keywords:** 综合性实验, 药物分析, 卤键有机框架材料, 分散固相萃取, 溴代阻燃剂, comprehensive experiment, pharmaceutical analysis, halogen-bonded organic frameworks, dispersive solid-phase extraction, brominated flame retardants

## Abstract

为适应新时代对药学人才创新与实践能力的高要求，贯彻“以学生为中心、以成果为导向”的教育理念，本教学团队对药物分析课程进行了深入改革，设计并实施了一项面向本科生的综合性、研究型教学实验。该实验以功能材料的设计与应用为主线，引导学生通过自主合成卤键有机框架材料（XOF-1），并系统完成其结构表征、性能研究与分析应用。实验聚焦环境分析中的实际问题，要求学生将合成的材料用作分散固相萃取吸附剂，用于选择性富集典型溴代阻燃剂（包括四溴双酚A、四氯双酚A和六溴苯），并实现实际水样中四溴双酚A的去除。教学环节完整覆盖“材料制备、结构表征、方法开发和性能评价”的全流程，使学生亲历从材料设计到实际应用的完整科研训练路径，深度体验“理论-设计-实验-分析”的科学研究逻辑。本实验使学生扎实掌握了功能材料的合成、表征及其在样品前处理中的应用技能，并在方案设计、条件优化与结果分析中，系统提升了批判性思维、问题解决和自主探究能力。该实践有效激发了学生对科研的兴趣，强化了学生的创新意识，增强了学生在环保与公共健康领域的使命感，实现了知识、能力与价值培养的有机统一，达到了实验教学全方位育人的目标。

## Abstract (en)

To address the evolving demands for innovation and practical competencies in pharmaceutical education， and in alignment with the “student-centered， outcome-driven” pedagogical approach， our teaching team has undertaken a comprehensive reform of the Pharmaceutical Analysis curriculum. We designed and implemented an integrative， research-based laboratory experiment for undergraduate students. Centered on the design and application of functional materials， the experiment guides students through the independent synthesis of a halogen-bonded organic framework， designated as XOF-1， followed by systematic structural characterization， performance evaluation， and analytical application. The experiment is grounded in real-world environmental analysis challenges， requiring students to employ the synthesized material as a dispersive solid-phase extraction （DSPE） adsorbent for the selective enrichment of typical brominated flame retardants， including tetrabromobisphenol A （TBBPA）， tetrachlorobisphenol A （TCBPA）， and hexabromobenzene （HBB）， and to achieve the removal of TBBPA from actual water samples. The instructional process fully encompasses the workflow from material preparation and structural characterization to method development and performance assessment， enabling students to experience a complete research pathway from material design to practical application. This approach immerses them in the scientific inquiry cycle of theory-design-experiment-analysis. Through this experiment， students gain solid proficiency in synthesizing and characterizing functional materials and applying them to sample pretreatment. By engaging in experimental design， condition optimization， and data interpretation， they develop critical thinking， problem-solving skills， and capacity for independent inquiry. The exercise fosters deep integration of theoretical knowledge and practical skills， stimulates research interest， strengthens innovative and applied mindsets， and cultivates a sense of professional responsibility in environmental and public health. Ultimately， it achieves a cohesive blend of knowledge acquisition， competency development， and value cultivation， fulfilling the overarching goal of holistic education through experimental learning.

药物分析作为一门聚焦药品质量研究与控制的学科，其教学目标在于培养学生建立系统的质量观念，掌握从原料到制剂的全流程分析评价能力。在这一体系中，样品前处理是决定分析结果准确性与可靠性的关键环节，它如同连接复杂实际样品与现代精密仪器的“桥梁”，其核心在于通过净化、富集与形式转化，实现目标物的选择性提取与干扰基质的高效去除，从而保障检测方法的灵敏度、选择性及仪器系统的稳定运行^［1］^。在药物研发、生产质控、临床监测等实际应用中，规范、高效的样品前处理是确保数据合规、支撑科学决策的重要基础。为推动教学与科研深度融合，本教学团队依托在有机框架材料及其样品前处理新方法方面的研究积累，将前沿科学问题转化为本科综合性实验教学资源，设计了相应的综合性实验，以激发学生的科研兴趣，提升实验操作能力，并培养其创新与实践素养。

分散固相萃取（DSPE）作为一种高效、简便的样品前处理技术，具有操作简单、萃取速度快、溶剂消耗少、便于自动化等优点^［2］^。该技术的核心在于吸附剂的选择，其性能直接影响目标物富集效率与方法的选择性^［3］^。理想的吸附剂应具备高比表面积、可调控的孔道结构、丰富的活性位点及良好的化学稳定性，以实现对目标物的高吸附容量与高选择性吸附。目前，一系列先进多孔材料已被广泛应用于分散固相萃取中，包括金属有机框架（metal-organic frameworks，MOFs）^［4，5］^、共价有机框架（covalent organic frameworks，COFs）^［6-8］^、多孔有机聚合物（porous organic polymers，POPs）^［9，10］^等。这些材料凭借其独特的结构特性，在复杂基质样品前处理中表现出良好的应用潜力。然而，它们在面向特定分析物时仍存在一定局限性，例如在复杂基质中的选择性识别能力不足、功能性修饰手段有限等问题，从而限制了其在实际样品中的广泛应用。

卤键有机框架材料（halogen-bonded organic frameworks，XOFs）是一类通过卤键（X…B）作用构筑的新型多孔晶体材料。其结构中作为电子受体的卤原子，可与含氮、氧等富电子官能团的目标分析物之间形成具有高方向性与特异性的卤键相互作用，从而表现出优异的选择性识别与吸附能力^［11，12］^。该类材料不仅拥有结构明确且可设计的多孔框架，其卤键功能位点亦易于理性调控。这种独特的双重优势使其在复杂基质中对痕量目标物表现出极高的选择性富集能力，因而成为构建新一代高选择性样品前处理方法的理想材料^［13，14］^。然而，截至目前，基于XOFs的DSPE新方法仍鲜有报道。此外，溴代阻燃剂如四溴双酚A（TBBPA）在环境水体中普遍存在且浓度较低，具有持久性、生物蓄积性和潜在内分泌干扰性等特征，可对水生生态系统和人体健康构成长期风险^［15，16］^，因而开发能够实现该类痕量污染物选择性富集与去除的方法具有重要的科学意义与应用价值。针对上述问题，本实验以典型溴代阻燃剂为目标分析物，设计并合成对其具有选择性富集能力的XOF（命名为XOF-1）。首先，对所得材料进行系统的物理化学表征；随后将其作为DSPE吸附剂，优化萃取条件；最终建立基于XOF-1的DSPE与高效液相色谱（HPLC）联用的分析方法，并应用于实际水样中目标分析物的检测，为复杂基质中痕量溴代阻燃剂的选择性富集与分析提供新材料与新方法支持。

基于药物分析学科实践性强、应用性广的特点，本科阶段的药物分析综合实验应注重引导学生理论联系实际，激发学习主动性与科研创新思维。目前本校已开设若干覆盖经典实验内容的基础性教学实验，但在前沿性与创新性方面仍显不足，尤其样品前处理模块主要停留在蛋白沉淀、液液萃取等传统方法。为此，本课程引入“基于新材料的DSPE新方法开发”作为本科药物分析综合实验的教学项目，将新型吸附剂的制备、结构表征、性能评价与实际应用有机融入教学全过程，涵盖有机化学、分析化学、仪器分析等多学科交叉知识。本实验设计旨在实现以下目的：（1）掌握XOFs材料的设计合成与表征技术，理解其基于卤键作用的选择性吸附机制；（2）系统学习DSPE方法的建立与优化，掌握吸附剂用量、萃取时间、洗脱条件等关键参数的考察方法；（3）建立XOFs-DSPE与HPLC联用分析方法，实现实际水样中痕量目标溴代阻燃剂的检测，以提升综合实验设计与分析问题的能力。其核心原理与理念如下：XOFs是一类通过卤键（X…B）作用构筑的多孔晶体材料，结构中卤原子（如Br、I）作为电子受体，可与含富电子官能团的溴代阻燃剂（如TBBPA）形成X…X、X…*π*、X…O等高方向性和特异性的卤键相互作用，实现对目标物的高选择性识别与富集；DSPE利用吸附剂与样品溶液直接分散接触，增大接触面积，通过离心或分离后洗脱，实现快速、高效、低溶剂消耗的萃取净化；同时，采用系统表征手段表征材料结构与形貌，并在参数优化后与HPLC联用，实现复杂水样中痕量溴代阻燃剂的灵敏检测。本实验通过“材料设计-合成表征-方法建立-实际应用”的全流程训练，强化学生对样品前处理新技术的理解，整合多学科知识，旨在有效提升学生的知识整合能力、综合运用能力与科研探索精神，为后续实验部分的开展奠定基础。

## 1 实验部分

### 1.1 仪器、材料与试剂

Empyrean多功能高分辨X射线衍射仪（XRD，荷兰帕纳科公司）；IR Affinity-1S傅里叶变换红外光谱仪（FT-IR，日本Shimadzu公司）；Zetasizer Nano ZS90电位与粒度分析仪（英国Malvern公司）；FCA 2000A AFES接触角测量仪（中国爱飞思公司）；AVANCE III 400核磁共振波谱仪（NMR，德国Bruker公司）；安捷伦1260高效液相色谱仪（HPLC，美国安捷伦公司）；Apreo 2C扫描电子显微镜（SEM）、Talos F200S G2透射电子显微镜（TEM）及K-Alpha+ X射线光电子能谱仪（XPS）（美国Thermo Fisher Scientific公司）。

1，3，5-三（吡啶-4-基）苯（TPy，>97%）购自上海吉至生化科技有限公司；三氟甲烷磺酸银（AgOTf，>99%）购自上海阿拉丁生化科技股份有限公司；TBBPA（>98%）、四氯双酚A（TCBPA，>98%）和六溴苯（HBB，>98%）购自上海麦克林有限公司；Br_2_、三氯甲烷（CHCl_3_）、四氢呋喃（THF）、甲醇（MeOH）等购自成都金山化学试剂有限公司，实验中所用水均为去离子水。所有化学试剂均为分析纯或更高纯度，无需进一步纯化处理。自来水样品采自西南医科大学药物分析实验室；池塘水样品采自西南医科大学校园池塘；沱江水样品采自泸州市沱江三桥段。

### 1.2 XOF-1的制备

XOF-1的合成参照文献［^12^］并作适当优化。具体步骤如下：将AgOTf（15.42 mg）溶于0.6 mL甲醇中，超声分散均匀。在搅拌下将该溶液逐滴加入4.0 mL含TPy（12.36 mg）的CHCl_3_-MeOH（4:1，体积比）中，室温搅拌1 h。随后，移取3 μL Br_2_加入3 mL CHCl_3_中，然后逐滴加入上述反应液，继续搅拌1 h。反应结束后，以7 600 r/min离心5 min，弃去上清液。所得沉淀转移至含有3.0 mL CHCl_3_-MeOH（3:1，体积比）的圆底烧瓶中，在N_2_气氛保护下于130 ℃搅拌反应5 h。反应完成后，再次以7 600 r/min离心5 min，沉淀用CH_2_Cl_2_洗涤3次，并于60 ℃真空干燥24 h，最终得到灰白色粉末产物XOF-1。产物保存于N_2_气氛中。

### 1.3 吸附等温线和吸附动力学研究

采用吸附等温线和吸附动力学实验对XOF-1的吸附性能进行系统研究。将HBB、TBBPA及TCBPA（结构式如图1所示）分别溶解于THF中，配制成质量浓度为10 mg/mL的储备液。随后，采用THF-水（4:1，体积比）稀释至10~800 µg/mL，供后续实验使用。

**图1:**
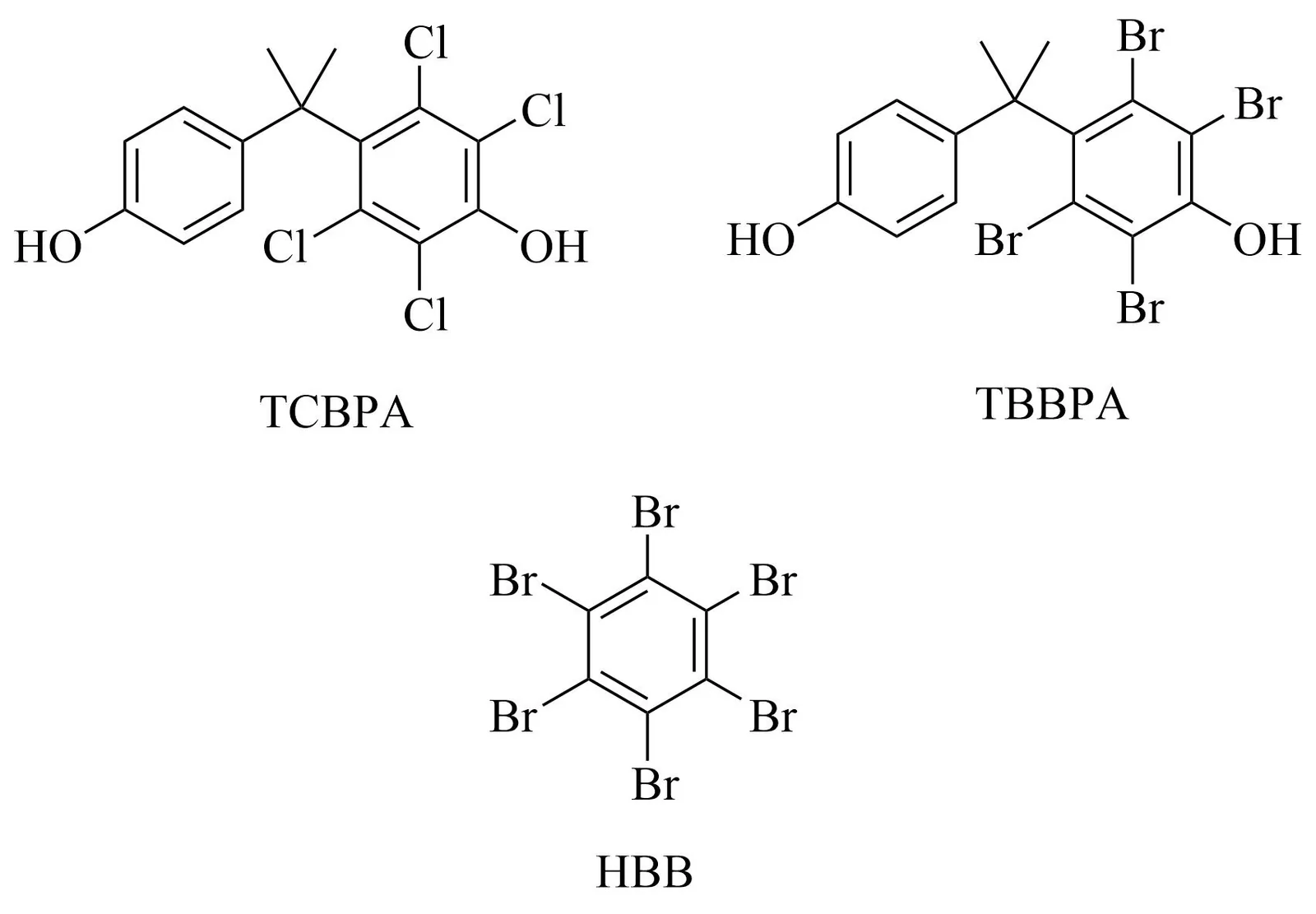
TBBPA、TCBPA和HBB的结构式

吸附等温线实验：分别称取材料约1 mg，置于含有不同质量浓度（10~800 µg/mL）目标物的1 mL工作溶液中，超声分散1 min后，置于恒温振荡器中于25 ℃下振荡吸附3 h。吸附结束后，用0.22 µm微孔滤膜过滤，取滤液进行HPLC分析。利用式（1）对材料吸附目标物的吸附量（*Q*，mg/g）进行计算：


$$Q=\frac{C_{0}-C_{e}}{m}\times V$$ （1）

式中，*C*_0_（μg/mL）为目标物初始质量浓度，*C*_e_（μg/mL）为吸附平衡时目标物的质量浓度，*V*（mL）为溶液体积，*m*（mg）为吸附剂用量。

吸附动力学实验：将约1 mg材料加入1 mL质量浓度为400 µg/mL的目标物溶液中，分别于不同时间点取样，其余操作步骤同吸附等温线实验。

### 1.4 样品采集及DSPE过程

为评价所建立方法的适用性，选用不同来源的实际水样进行实验，包括自来水、池塘水和江水。水样采集后经0.45 µm微孔滤膜过滤，去除悬浮颗粒物。由于这些样品中未检测到TBBPA，所有水样均采用标准添加方法，分别向3种实际水样中添加低、中、高3个水平（10、25、50 µg/mL）的TBBPA工作溶液，每个水平平行测定3次。按以下DSPE步骤对样品进行前处理：将1.5 mg的XOF-1加入含目标成分（TBBPA）的1 mL实际水样中，调节pH至7.0，涡旋1 min，4 000 r/min离心5 min，弃去上清液；向吸附剂中加入1.0 mL甲醇，振摇洗脱15 min，收集滤液，重复上述洗脱步骤3次，合并滤液，浓缩至1.0 mL，经0.45 μm滤膜过滤后进行HPLC分析。实验设计示意图见图2。

**图2:**
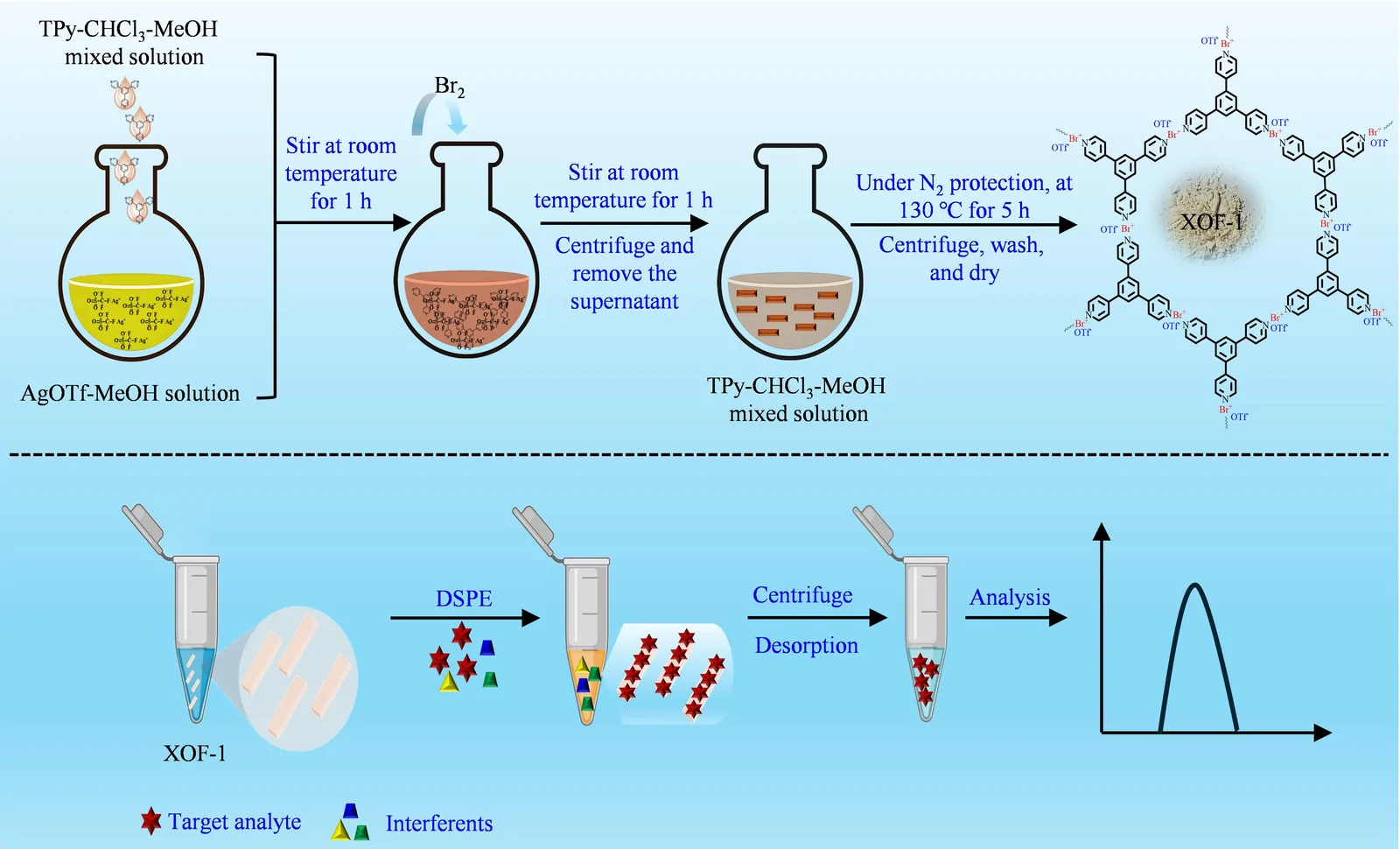
实验设计示意图

### 1.5 HPLC分析条件

色谱柱为安捷伦SB-C18反相柱（250 mm×4.6 mm， 5 µm）；流动相为0.01%甲酸水溶液-乙腈（40:60，体积比），采用等度洗脱模式；流速为1.0 mL/min；柱温为30 ℃；进样体积为5 µL；检测器为二极管阵列检测器，检测波长设定为210 nm。

## 2 结果与讨论

### 2.1 材料的表征

首先，采用XRD对XOF-1的晶体结构进行表征。结果如图3所示，XOF-1在2*θ*=5.05°、6.52°、7.50°、8.21°、9.64°和10.13°处出现明显的衍射峰，分别对应（110）、（001）、（011）、（120）、（021）和（300）晶面。该衍射图谱与文献［^12^］报道一致，表明XOF-1具有有序的晶体框架结构。

**图3:**
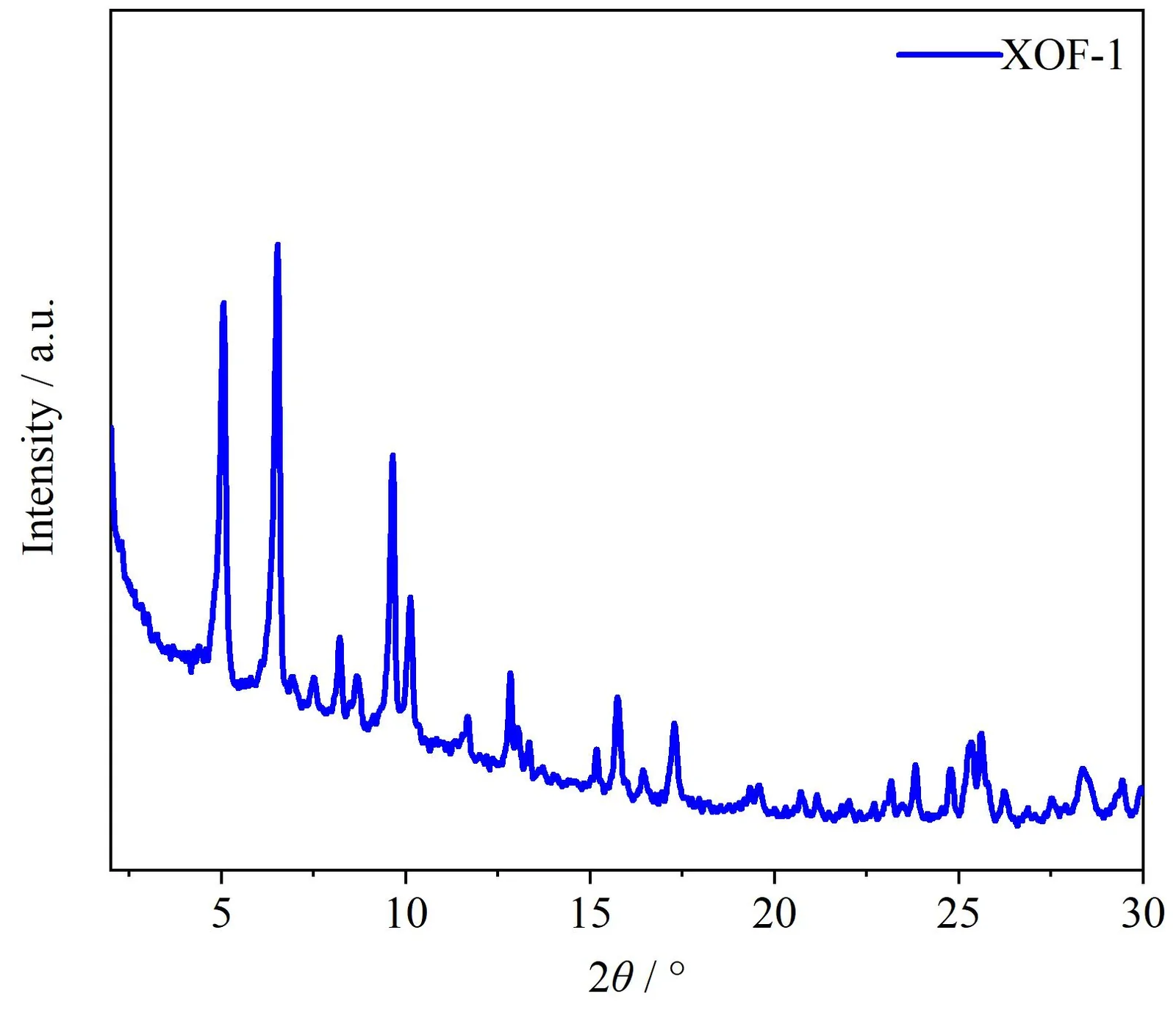
XOF-1的XRD图

SEM分析结果（图4a）显示，XOF-1呈现典型的纤维状微观形貌，其纤维结构均一、取向交错，相互交织形成具有开放孔道的三维网状结构。TEM图像（图4b）进一步表明，该纤维表面光滑、直径分布均匀，长度可达数微米。这种密集且相互贯通的纤维网络结构有利于暴露丰富的表面活性位点，为分析物的传质提供有效通道，在吸附等应用中展现出结构优势。

**图4:**
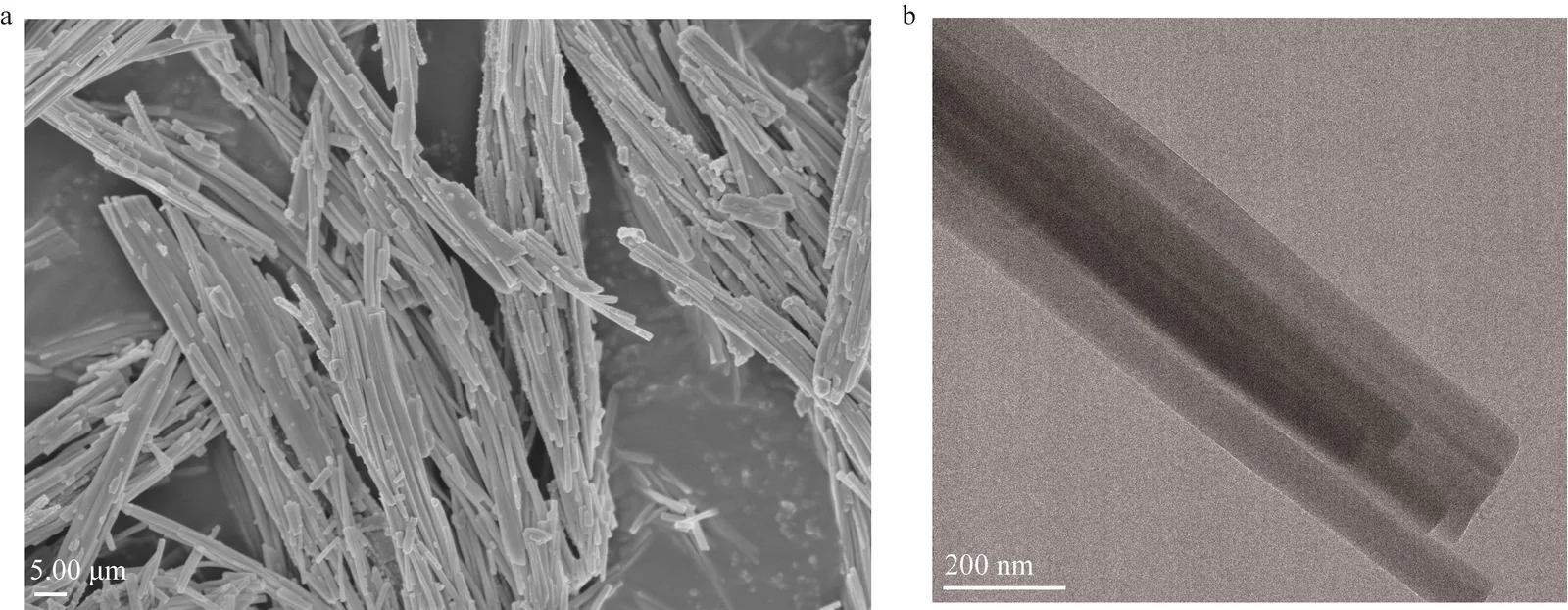
XOF-1的（a）SEM图和（b）TEM图

为了进一步确认XOF-1的官能团特性，采用FT-IR和^1^H NMR进行分析。如图5a所示，与TPy的FT-IR谱图相比，XOF-1的=C-H与C=N伸缩振动峰向高波数发生了移动，发生这种现象的原因可能是吡啶氮原子与Br^+^形成卤键后，导致吡啶环电子云密度显著降低（去屏蔽效应）^［12］^。^1^H NMR分析结果（图5b）显示，在TPy单体^1^H NMR谱图中，吡啶环质子信号分别位于*δ*=8.71~8.73（H_a_）、8.00~8.02（H_b_）和8.29（H_c_），峰形多重分裂。形成XOF-1框架后，谱图呈现明显变化：吡啶质子峰整体向低场移动（H_a_： 8.97~8.98， H_b_： 8.48~8.50， H_c_： 8.63），表明吡啶氮原子与Br^+^配位后电子云密度显著降低；同时峰强度显著增强、峰形趋于对称，反映出TPy单元在框架中大量、规则排列；溶剂峰相对比例相应减弱，进一步印证了高密度晶态结构的形成。这些变化证实了N…Br^+^…N卤键的成功构筑。

**图5:**
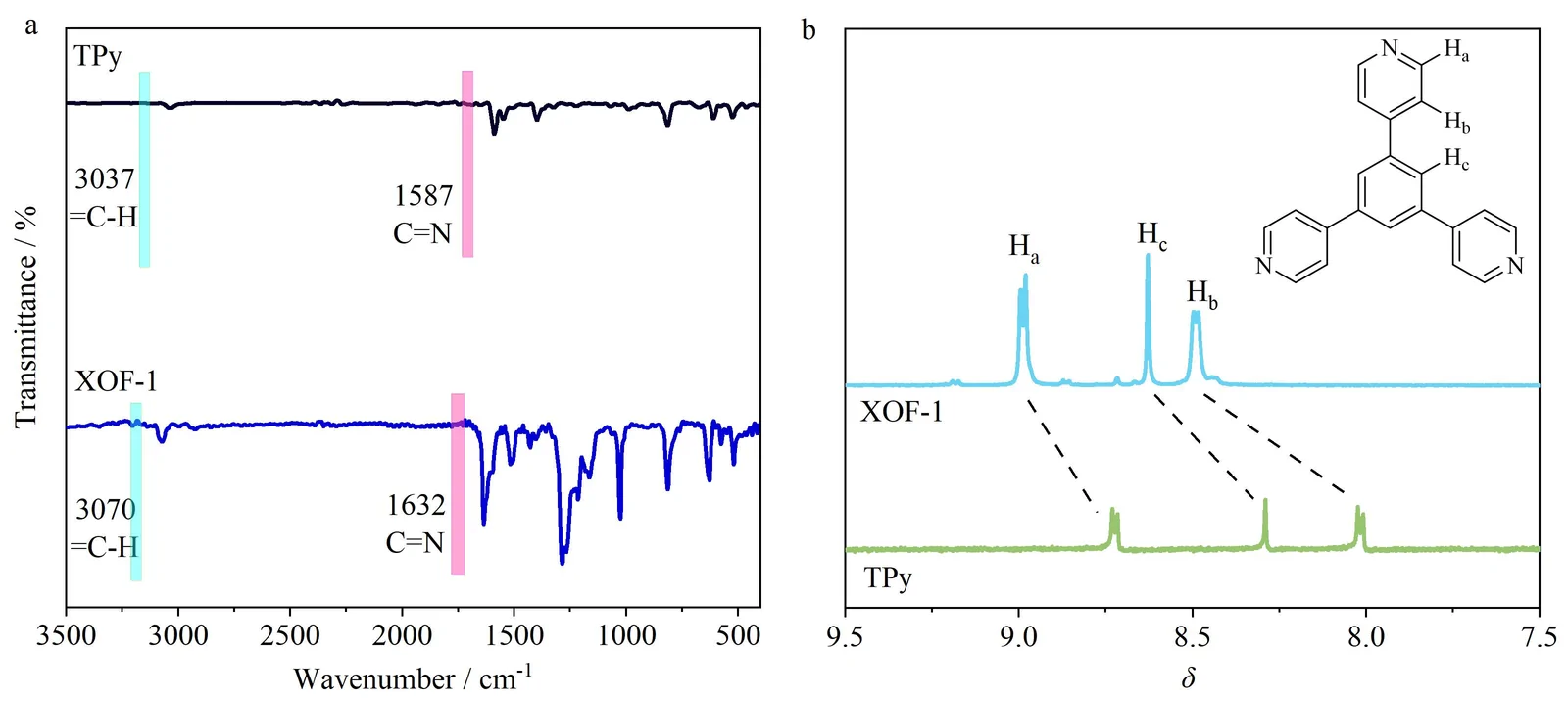
TPy和XOF-1的（a）FT-IR谱图和（b）^1^H NMR谱图

随后，采用热重分析和Zeta电位分析实验分别评估了材料的热稳定性和表面电荷特性。如图6a所示，XOF-1在360 ℃之前仅发生约4.30%的微量失重，主要归因于材料孔道内残留溶剂或表面吸附水的脱除；当温度超过360 ℃后，有机骨架开始显著分解，最终在900 ℃时剩余质量为55.82%。该结果表明XOF-1在360 ℃以下具有良好的热稳定性，能够满足多种高温或温和热过程的应用需求。

**图6:**
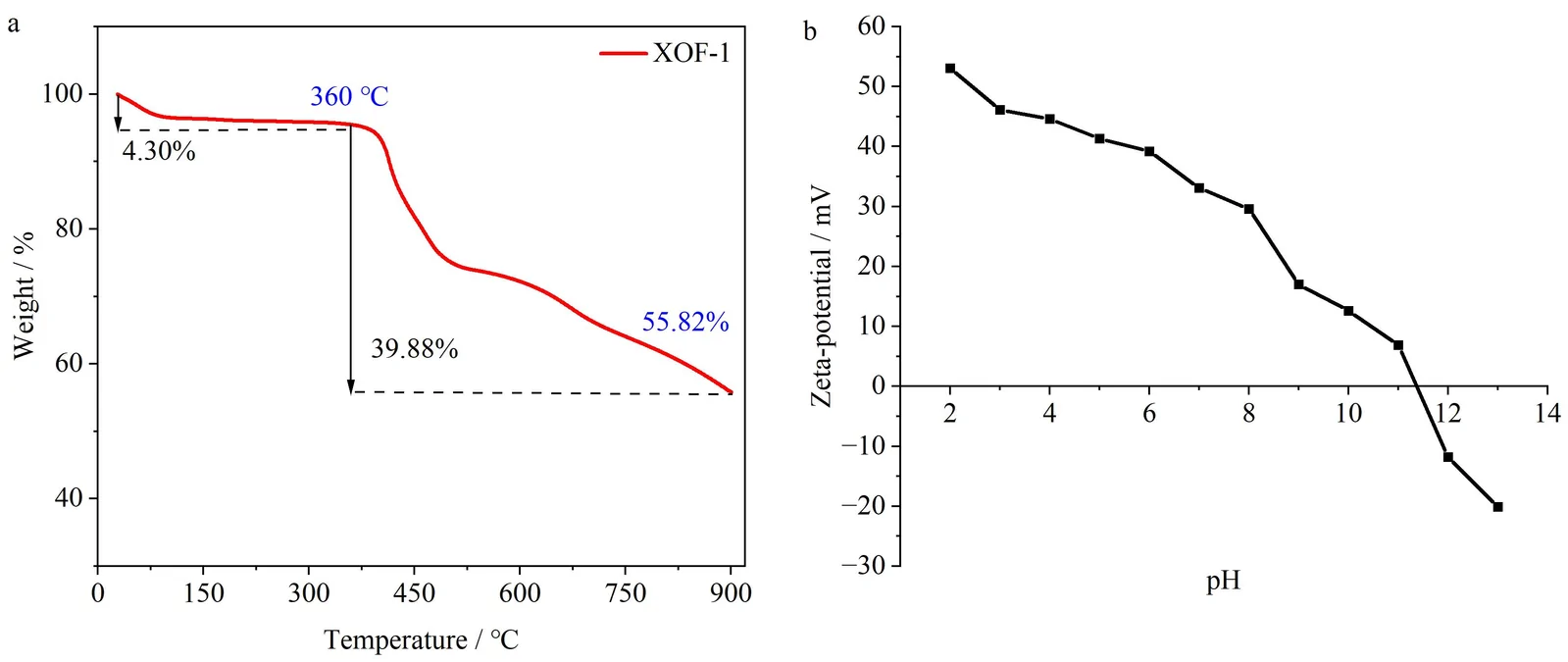
XOF-1的（a）TGA曲线和（b）不同pH条件下的Zeta电位图

此外，Zeta电位结果如图6b所示。XOF-1的等电点位于pH≈11.36，表明在接近中性和酸性条件下材料表面整体带正电荷；当体系pH<11.36时，材料表面带正电；当pH>11.36时，表面则带负电。这一特性意味着可通过调节溶液pH值有效调控材料表面电荷状态，进而影响其与带电分子、离子或污染物的静电相互作用，在选择性吸附、电催化、离子交换及功能性界面组装等应用中具有重要的调控意义。

最后，采用静态接触角测试对材料表面润湿性进行表征，结果如图7所示。XOF-1表面的水接触角约为29.30°，远小于90°，表明其具有显著的亲水表面特性。该特性主要源于材料骨架中吡啶氮原子及卤键（N…Br^+^）等极性位点对水分子的强相互作用。XOF-1的强亲水性有助于其在水相中迅速分散，进而提高与目标分析物的传质效率，从而优化萃取性能。

**图7:**
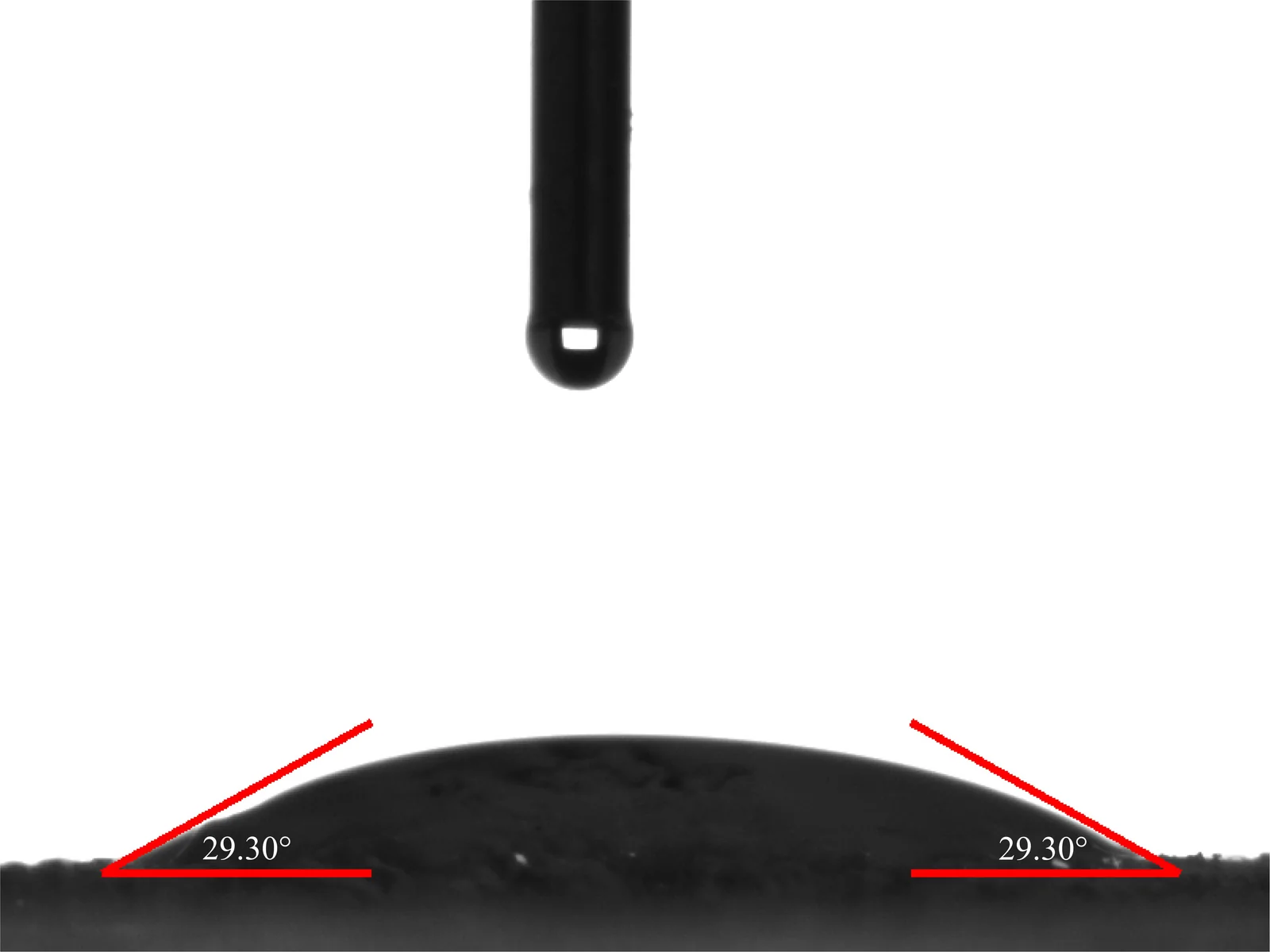
XOF-1的水接触角图

### 2.2 吸附性能评价

#### 2.2.1 吸附等温线评价

为了评估XOF-1对所选分析物的吸附性能，首先进行了吸附等温线评价。图8a展示了TBBPA、TCBPA和HBB 3种分析物在XOF-1材料上的吸附容量随初始浓度的变化规律。所有吸附曲线均呈现典型的“先快速上升后趋于饱和的”趋势，符合单分子层吸附平衡特征。其中，TBBPA的*Q*最高（约21 mg/g），而HBB最低（约10 mg/g）。推测原因：XOF-1对3种分析物的吸附差异主要源于其与材料特异性作用的强度不同。TBBPA吸附容量最高，是因为其分子同时具备强卤键供体（高极化率的溴原子）和氢键供体（酚羟基），能与XOF-1骨架中的吡啶氮和OTf^-^单元形成多重、协同的卤键和氢键作用，实现高效锚定。而TCBPA的氯原子卤键能力较弱，故吸附量次之；HBB分子高度对称且缺乏极性官能团，仅能依靠较弱的疏水作用和微弱的卤键吸附，因此吸附容量最低。

**图8:**
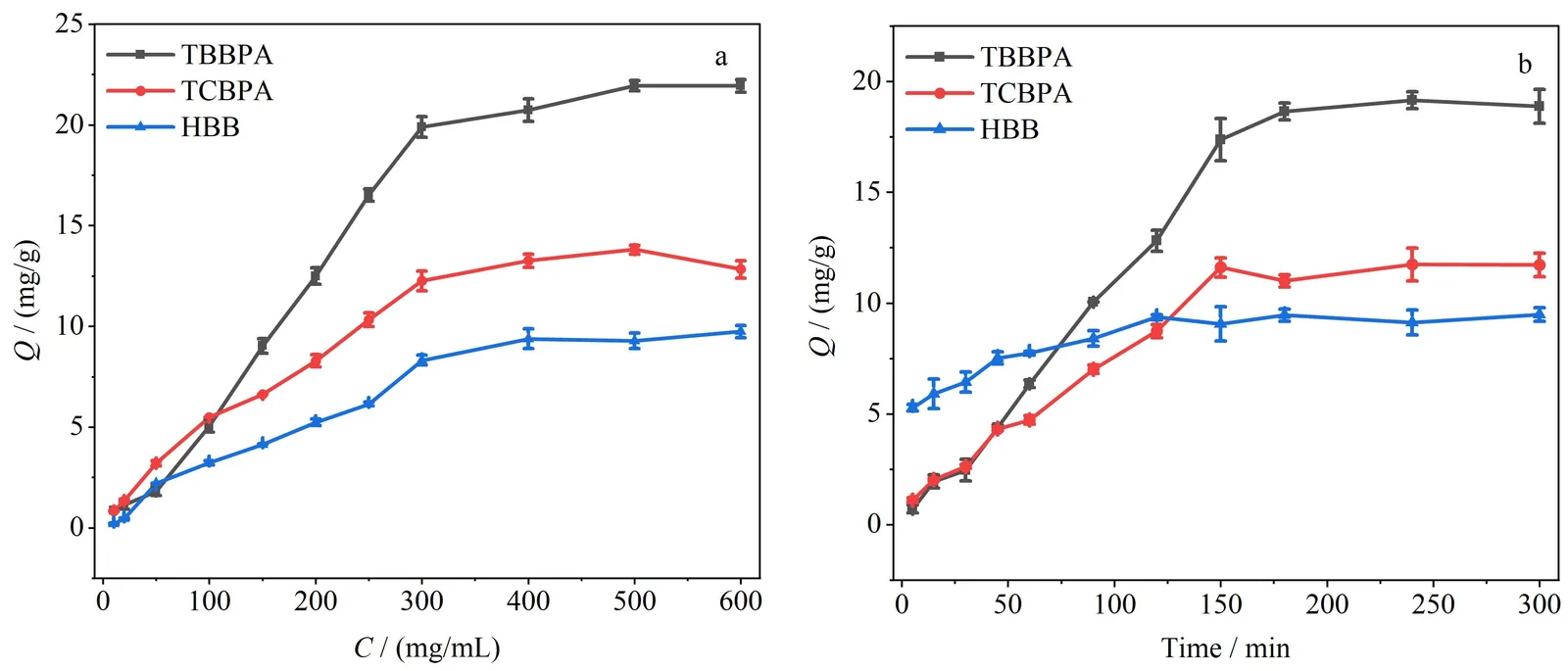
3种目标分析物的（a）吸附等温线和（b）吸附动力学曲线（*n*=3）

#### 2.2.2 吸附动力学评价

图8b展示了XOF-1对3种分析物（400 µg/mL）的吸附动力学行为。3种目标物均表现为典型的“快速吸附-逐步饱和”趋势，但达到平衡的时间存在差异：TBBPA和TCBPA约在150 min后趋于稳定，而HBB在120 min即基本达到平衡。在吸附容量方面，TBBPA最高，TCBPA次之，HBB最低，形成明确的TBBPA>TCBPA>HBB的吸附能力顺序。这一结果与分子-材料间的相互作用机制高度吻合：TBBPA同时具备强卤键供体（Br）与氢键供体（-OH），能与XOF-1骨架中的吡啶氮和OTf^-^单元形成卤键、氢键等多重协同作用，从而表现出最强的吸附亲和力与最高的平衡吸附量；TCBPA虽具类似结构，但Cl原子的卤键能力较弱，故吸附容量较低；而HBB分子高度对称、缺乏极性官能团，主要依赖较弱的疏水作用与XOF-1结合，因此吸附容量最小且更快达到饱和。

### 2.3 DSPE条件优化

如前所述，XOF-1材料对TBBPA表现出优异的吸附性能，可作为一种具有应用潜力的选择性吸附剂。在实际使用中，其吸附效率和循环再生能力受多种参数影响，包括吸附剂用量、盐浓度、pH、解吸溶剂的类型、解吸时间及解吸次数等。为了获得最佳使用性能，需对这些参数进行系统优化。优化实验均在200 μg/mL的TBBPA水平下进行，每组实验平行测定3次。

#### 2.3.1 吸附剂用量的优化

考虑到XOF-1的用量显著影响其对TBBPA的吸附效果，本文系统研究了不同XOF-1用量下的吸附性能。结果如图9a所示，随着XOF-1用量从0.5 mg增至2.5 mg，其对TBBPA的吸附效率逐渐提升，并在吸附剂用量为1.5 mg时趋于平衡。因此，在后续实际应用中选择1.5 mg作为吸附剂用量。

**图9:**
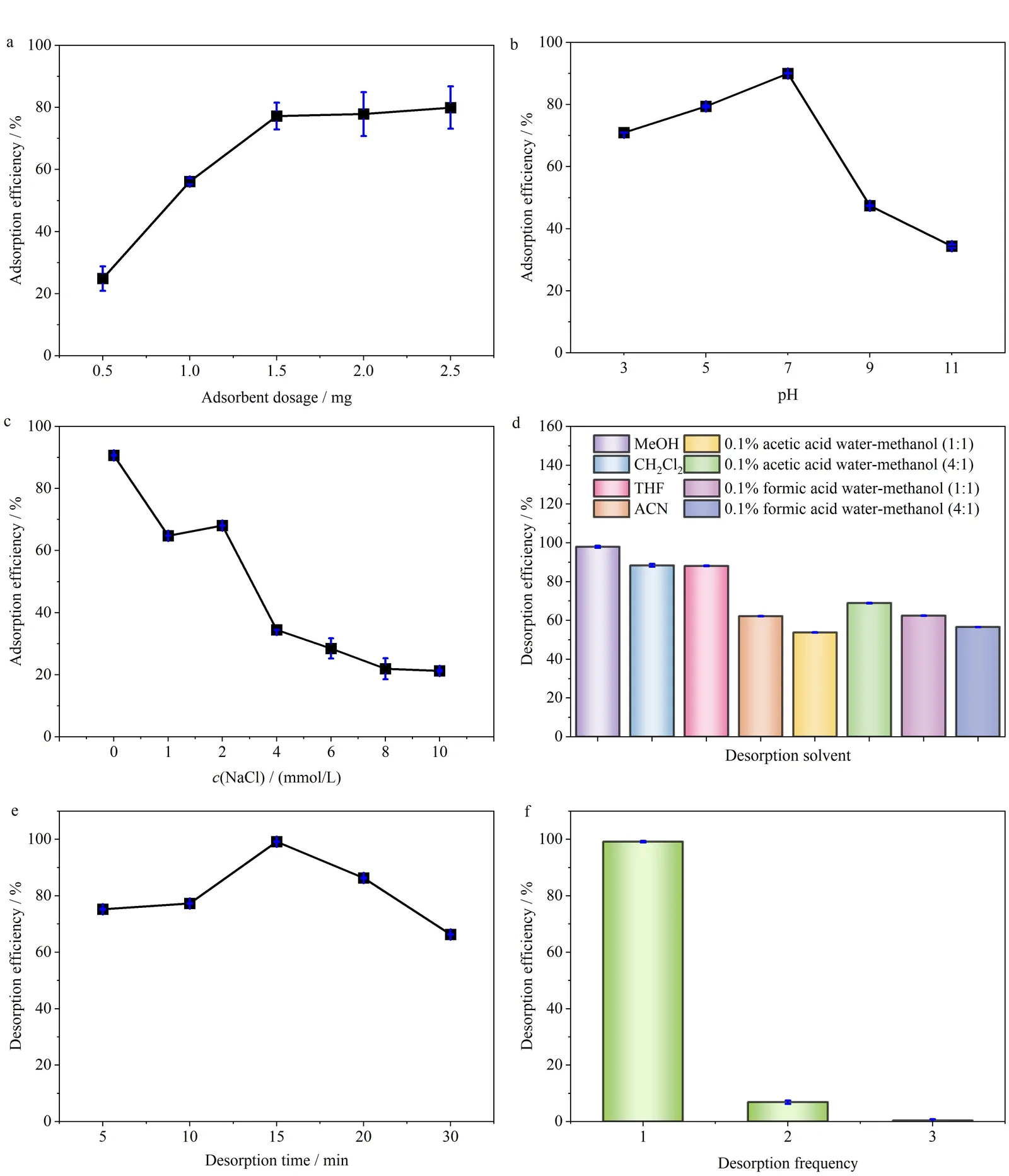
（a）吸附剂用量、（b）pH和（c）NaCl浓度对XOF-1吸附效率的影响；（d）解吸溶剂种类、（e）解吸时间和（f）解吸次数对XOF-1解吸效率的影响（*n*=3）

#### 2.3.2 pH的影响

溶液的pH是影响XOF-1吸附行为的关键因素之一。实验考察了不同pH条件下XOF-1对TBBPA的吸附效率，结果如图9b所示。在pH 3~7范围内，随着pH升高，吸附效率逐渐上升，在pH≈7时达到峰值；当pH>7时，吸附效率则逐渐下降。该变化趋势与吸附剂表面电荷、污染物存在形态及两者间的相互作用机制密切相关。XOF-1的零点位为11.36，当pH<11.36时材料表面带正电，反之则带负电。TBBPA为二元酚类化合物，p*K*_a_分别约为7.5与8.5，在pH 7.5时主要以中性分子形式存在，而在pH 8.5时主要转化为阴离子形式。在pH 3~7 范围内，XOF-1表面带正电，若仅从静电作用判断应不利于吸附，但该范围内的吸附效率并未降低，反而逐渐升高。其原因在于，随着pH向中性靠近，H^+^浓度降低，减少了H⁺与目标物在吸附位点上的竞争，同时材料与污染物之间的非静电作用逐步显现。其中，卤键作用尤为重要：在近中性条件下，TBBPA中的溴原子可作为卤键供体，与XOF-1表面的富电子区域形成具有高方向性和较强作用力的卤键，部分抵消了静电排斥的负面影响，从而提升了吸附效率。当pH处于7~11范围内时，TBBPA转变为阴离子形态，XOF-1表面仍带正电，二者之间存在静电吸引，理论上有利于吸附。然而实际吸附效率在pH>7后呈下降趋势。这主要是因为随着pH升高，TBBPA去质子化削弱了其*σ*-hole特征，导致其作为卤键供体的能力降低；同时溶液中大量OH^–^会与目标物竞争吸附位点，进一步抑制了吸附过程。综上，在pH≈7时吸附效率最高，不仅是由于静电排斥相对较弱、H^+^竞争减弱，更得益于卤键作用在该条件下的显著贡献。卤键作为一种高选择性的分子识别机制，有效增强了XOF-1在近中性环境中对TBBPA的吸附能力，是理解其吸附行为的重要机制之一。综上，在后续DSPE过程中将设定pH为7。

#### 2.3.3 NaCl的影响

图9c显示，随着NaCl浓度的升高，XOF-1对TBBPA的吸附效率呈下降趋势。在0 mmol/L的NaCl浓度下，吸附效率达到最高（约90%），而当浓度增加至10 mmol/L时，吸附效率明显降低。这种变化可能是由于盐离子对吸附过程的双重影响：一方面，较高的离子强度会压缩双电层，削弱XOF-1表面与TBBPA之间的静电相互作用；另一方面，Na⁺和Cl⁻离子会竞争占据XOF-1表面的吸附位点，从而抑制目标污染物的有效吸附。综上，在后续的DSPE过程中将不添加NaCl。

#### 2.3.4 解吸附条件的优化

为有效解吸XOF-1上吸附的TBBPA，本研究系统筛选了多种溶剂。图9d所示结果表明，单一甲醇体系呈现出最佳解吸附效率，表明甲醇对TBBPA具有较强的亲和力，可有效破坏TBBPA与XOF-1之间的非共价相互作用（如卤键、氢键、*π-π*堆积等），实现目标物的有效释放。与混合溶剂相比，甲醇组成简单，避免了多元体系中可能存在的溶剂协同或竞争效应，有利于解吸过程的可控性与重复性。在此基础上，优化了解吸时间参数。图9e结果显示，在15 min时解吸效率达到稳定，继续延长时间未见明显提升，因此确定最佳解吸时间为15 min。此外，为确定实现TBBPA完全解吸所需的条件，进一步考察了解吸次数对洗脱效果的影响。图9f表明，经3次连续解吸操作后，XOF-1上吸附的TBBPA几乎全部洗脱，故确定最佳解吸次数为3次。

### 2.4 实际样本分析

基于对自来水、池塘水和江水的加标实验结果（见表1），XOF-1对TBBPA的吸附回收率为60%~90%，且所有样品测定结果的RSD均≤4.93%，表明该材料具有可靠的富集性能和良好的实验重复性。在较低质量浓度（10 µg/mL）条件下，3种实际水样中的回收率均位于70%~85%范围内。其中，自来水与江水的回收率相对较高（分别为82.19%和83.86%），而池塘水的回收率略低（74.66%），这可能与池塘水中含有较多的天然有机物、悬浮颗粒等竞争性杂质有关，但整体回收率仍处于可接受范围内。随着加标质量浓度提高至25 µg/mL和50 µg/mL，吸附回收率仍可稳定维持在60%以上，且RSD进一步降低（例如池塘水50 µg/mL实验组的RSD低至0.32%），说明在较高目标物浓度下，富集与检测过程的精密度有所改善。该性能主要源于XOF-1对TBBPA的卤键特异性识别作用，以及所优化的吸附-解吸条件使其在复杂基质环境中仍可保持结构稳定性和吸附选择性。综上所述，XOF-1在多种实际水样中均展现出良好的富集效率和抗干扰能力，结合HPLC的检测方法，为环境中痕量溴代阻燃剂的灵敏、可靠分析提供了有效的技术支持。

**表1:** XOF-1存在下TBBPA在实际水样中的吸附回收率（*n*=3）

Sample	Initial/（μg/mL）	Final/（μg/mL）	Recovery/%	RSD/%
Tap water	9.46	7.76	82.19	4.93
	25.01	18.27	73.06	0.84
	52.97	32.52	61.39	1.16
Pond water	8.80	6.57	74.66	1.56
	25.72	22.31	86.74	4.27
	52.10	36.27	69.62	0.32
River water	10.05	8.43	83.86	1.01
	21.02	14.38	68.42	1.21
	51.50	31.88	61.91	2.22

Samples were spiked at three levels： 10， 25， and 50 μg/mL.

## 3 实验安排与教学反思

### 3.1 实验教学安排

基于上述实验设计与教学实践，本综合性实验共设计为16学时，分为课前预习、课堂实践操作、数据分析与报告撰写3个阶段：第1~2学时（课前）学生通过线上平台预习文献与安全须知，完成预习报告并提出科学问题；第3~14学时（课堂）依次完成XOF-1的合成与表征（4学时）、DSPE方法建立与优化（4学时）以及实际水样加标回收与HPLC检测（4学时）；第15~16学时（课后）学生处理数据、绘制图表并撰写包含原理、步骤、结果讨论与反思的综合实验报告。

### 3.2 教学反思

#### 3.2.1 教学成效

本实验以“卤键有机框架材料的设计-合成-表征-应用”为主线，将材料化学、分析化学与仪器分析等多学科知识有机融合。通过“文献调研-实验设计-操作实践-数据分析”全流程训练，学生不仅掌握了XOFs的合成与表征技能、DSPE方法建立与优化策略，还显著增强了对新材料、新方法在复杂样品前处理中应用价值的理解。多数学生能够主动对比TBBPA与其他污染物的吸附差异，并从分子结构、卤键作用等角度进行机制探讨，体现了科研思维与综合分析能力的提升。

#### 3.2.2 存在问题与改进方向

表征设备不足：XRD、XPS等大型仪器机时紧张，只有少部分学生可实际操作。未来可引入虚拟仿真教学模块，提前训练仪器操作与数据分析流程。实验周期较长：XOF-1合成与吸附动力学实验耗时较多，可能导致课堂时间紧张。后续可以将部分吸附平衡实验调整为课前演示或视频学习，集中课堂时间用于关键步骤操作与现象观察。前沿性延伸不足：目前实验聚焦于溴代阻燃剂，可鼓励学有余力的学生拓展至其他卤代污染物（如全氟化合物、药物残留），或尝试设计不同卤键受体（如碘代XOFs），进一步激发创新思维。

#### 3.2.3 综合评价

本实验将学科前沿（卤键有机框架）与经典分析方法（DSPE-HPLC）相结合，既夯实了学生的基本操作技能，又引入了新材料、新原理的研究视角，有效实现了“教学-科研”的深度融合。通过实际水样的加标回收分析，学生不仅掌握了方法建立与验证的全过程，更增强了解决复杂分析问题的信心与能力，为后续科研训练或专业实践奠定了扎实基础。

## 4 结语

本实验以《药物分析》课程改革为背景，设计并开展了一项面向新型样品前处理技术的综合性教学实验。通过引入具有明确结构功能特性的先进吸附材料（如XOF-1），结合现代分析仪器手段，引导学生深入理解材料合成、表征与应用之间的“构效关系”，并掌握液相色谱等设备的工作原理、操作流程与数据分析方法，逐步形成面向复杂基质开展方法设计与优化的系统性策略。本实验融合材料、分析、分离等多学科知识，形成贯穿“理论-实践-评估”的全流程教学模式，从文献调研、方案设计到实验操作、数据处理，再到报告撰写与结果讨论，全面提升学生的科学思维、实践能力与团队协作意识。教学实践通过理论与科研的深度融合，有效激发了学生的科研兴趣与创新意识，强化其在真实科学问题中发现、分析与解决复杂问题的能力。该实验为学生打下扎实的理论与实践基础，有助于培养兼具国际视野、创新能力与严谨科学精神的分析科学专业人才。本实验体现了“以学生为中心、以能力为导向”的教学理念，践行“教学与科研协同、理论与实践结合”的教育思路，为《药物分析》及相关学科开展综合性、设计性与探究性实验教学，提供了具有示范意义和参考价值的实施路径。

## References

[1] Jiang Y Y， Guo R L， Song Z Y， et al . Chinese Journal of Chromatography， 2026， 44（5）： 589 10.3724/SP.J.1123.2025.08009 PMC13150831 42093284

[2] Nie W D， Shuai S J， Hu K， et al . Chinese Journal of Chromatography， 2025， 43（6）： 678 10.3724/SP.J.1123.2024.07005 PMC12093210 40394747

[3] Felli N， Antonelli L， De Cesaris M G， et al . TrAC-Trends Anal Chem， 2026， 198： 118759

[4] Qin P G， Chen D K， Li D， et al . Food Chem， 2023， 409： 135272 10.1016/j.foodchem.2022.135272 36623357

[5] Hu Y Y， Wu B X， Tang Y S， et al . Anal Chim Acta， 2024， 1317： 342908 10.1016/j.aca.2024.342908 39030009

[6] Hao W L， Yin F H， Wang K， et al . Microchem J， 2024， 202： 110770

[7] Gao Y Y， Ding Y L， Chen L Y， et al . Chinese Journal of Chromatography， 2023， 41（7）： 545 10.3724/SP.J.1123.2022.12021 PMC10311619 37387275

[8] Sun Z Y， Yuan G L， Niu Y H， et al . Chinese Journal of Chromatography， 2026， 44（7）： 731 10.3724/SP.J.1123.2025.08002 PMC13352146 42421347

[9] He X Y， Lan W C， Lv Y C， et al . Chemosensors， 2026， 14（2）： 14020031

[10] Weng X， Liu S T， Huang J， et al . J Chromatogr A， 2024， 1717： 464707 10.1016/j.chroma.2024.464707 38310703

[11] Yang B H， Wang Y X， Liang J J， et al . Nat Commun， 2026， 17（1）： 1682 10.1038/s41467-026-68381-9 PMC12909867 41530182

[12] Bai X G， Tian Z N， Dong H Q， et al . Angew Chem Int Edit， 2024， 63（35）： e202408428 10.1002/anie.202408428 38847190

[13] Zhao Q， Sun P H， Gong G F， et al . Sci China Chem， 2025， 68（2）： 631

[14] Ji B M， Zhang D Y， Liang R， et al . Cryst Growth Des， 2021， 21（1）： 482

[15] Li M， Gong X Y， Tan Q W， et al . Sci Total Environ， 2024， 939： 173224 10.1016/j.scitotenv.2024.173224 38763187

[16] Zou Y M， Wang L C， Liu Q Y， et al . Sep Purif Technol， 2024， 329： 125169

